# Explaining the Concept of Self-Care Competence and Its Dimensions in Elderly Women with Knee Osteoarthritis in Iran: A Qualitative Study

**DOI:** 10.4314/ejhs.v33i1.19

**Published:** 2023-01

**Authors:** Faranak Kooranian, Zohreh ParsaYekta, Maryam Rassouli

**Affiliations:** 1 Department of Nursing, PhD Candidate of Nursing and Midwifery, Tehran Medical Sciences, Islamic Azad University, Tehran, Iran; 2 Department of Nursing, Faculty of Nursing and Midwifery, Tehran Medical Sciences, Islamic Azad University, Tehran, Iran; 3 Cancer Research Center, Shahid Beheshti University of Medical Sciences, Tehran, Iran

**Keywords:** Aged, Women, Osteoarthritis, Knee, Self Care, Qualitative Research

## Abstract

**Background:**

Chronic diseases, especially knee osteoarthritis, are more likely to occur among women with the increase in age. Self-care is an effective strategy for the management of disease in patients with knee osteoarthritis. Therefore, recognizing the dimensions of self-care competence in elderly women with knee osteoarthritis is particularly important for long-term management of the disease. The current study aimed to explain the concept and dimensions of self-care competence in elderly women with knee osteoarthritis.

**Methods:**

This qualitative study was conducted using a conventional content analysis method proposed by Graneheim and Landman, from March to November 2020 in Mashhad (one of the largest cities in Iran). A total of 19 participants including 11 elderly women with knee osteoarthritis, 4 first-degree relatives and 4 medical staff were selected by the purposive sampling. Data were collected through in-depth and semi-structured interviews which continued until data saturation was reached. The MAXQDA (Version 10) was used to organize, code, and manage the data.

**Findings:**

Three main themes including “symptom management”, “personal growth” and “social cohesion” were emerged as the dimensions of self-care competence in elderly women with knee osteoarthritis.

**Conclusions:**

Understanding the dimensions of self-care competence as one of the basic needs of the elderly women with knee osteoarthritis who live alone, is very important. Symptoms management, personal growth, and social cohesion as dimensions of self-care competence among this group of the elderly help to develop self-care competence interventions based on their needs.

## Introduction

Aging is the final part of the human life cycle and reaching this stage is a significant progress for humanity (1). According to the World Health Organization, 20% of the world's population will be over 60 years old by 2050 (2). The world's elderly population will be tripled by 2050, of which two-thirds live in developing countries (3). In 2020, the number of elderly people over 60 in Iran was 8.5 million. From 1977 to 2020, i.e. in 43 years, the elderly population of Iran increased from 5% to 10%, and then, in a surprising speed, which is unique in the world, this population will be increased from 10% to 20% only within 23 years (from 2020 to 2042). The continuation of this growth in the elderly population will cause this population to exceed one third of the country's population, so that by 2050, about 30%, and by 2057, about 33% of the country's population will be the elderly (4). Simultaneous with the increase in the number of elderly people in the country, studies show that the proportion of elderly women living alone is increasing more sharply compared to elderly men. The increase in the number of elderly people with the absolute celibacy and the single seniors (due to the increase in divorce during youth and middle age) cause an abrupt increase in the number of the alone or single elderly, especially lonely elderly women, in the next two decades (4). The growth of the elderly population significantly increases the need for long-term care (6). Also, living with a chronic disease has a significant impact on the level of competence, performance, quality of life and well-being of the elderly and has a great impact on productivity and health care costs in the society (7).

One of the chronic diseases that is the main cause of physical debility among the elderly is the knee osteoarthritis (8). Osteoarthritis of the knee is the most common debilitating joint disorder (9) and the most common musculoskeletal disease in people of 65 and older (10). It affects about 300 million people worldwide, and more than 40 million people in Europe. (11) Age is one of the most important risk factors for knee osteoarthritis and its prevalence is 85% between the ages of 75 and 79 (12). It is more prevalent among the women than men, especially after the age of 50 (9). This disease is associated with chronic inflammation and leads to damage to joint tissues including articular cartilage, subcutaneous bone, and synovium (9). Many people with knee osteoarthritis complain of vascular pain, stiffness and weakness, and difficulty in walking. Although knee osteoarthritis is a non-life-threatening disease, like other chronic diseases, it requires management of the daily activities at home and in the community, over a long period (13). These limitations can interfere with the daily activities of the individual (14). Therefore, it is raised as a general health challenge with significant consequences for vulnerable people, and imposes significant social and economic costs upon the health care systems. Self-care is an effective strategy for the management of disease in patients with knee osteoarthritis. Self-care can be defined as activities undertaken by individuals to promote their health, prevent disease, manage illness, and restore health as much as possible (9, 15–17). The potential and competence of older people for self-care in developed countries seems to be high, at least in terms of functional competence up to the age of 80 (15).

Competence refers to the growth of an individual's inner capacity to accept responsibility for life, self-control, self-efficacy, a sense of inner power, and a positive self-concept [16]. The basis of self-care competence is that human can act logically, has her own free will, and can act freely in relation to themselves and others. Self-care competence, which is considered as a potential competence, is a necessary condition for the realization of these measures. We can consider self-care competence as an individual's capacity for self-care. Self-care activities are viable when the individual has the potential for self-care competence (15). Since self-care competence is an individual's ability to recognize their needs, evaluate internal and external resources, and select self-care measures to achieve the desired goals of well-being and independence (1), it refers not only to functional ability, but also the cognitive and emotional component as well. As a process, self-care includes strategies and actions that enable s senior to modify or change their health status and subsequently adapt or expand their functional abilities. Therefore, self-care competence is a necessary condition for self-care activities in a way that leads to self-care actions (17). Achievement of a continuous care plan helps the elderly and their families to control and manage their disease at home (16–17). The results of previous studies on patients' competence showed that it has various dimensions that vary according to the nature of chronic disease, age, gender, environmental, and cultural context. To advance knowledge in these areas, current study explains the concept of self-care competence and its dimensions in elderly women with knee osteoarthritis. Elaborating the dimensions of self-care competence with a qualitative approach to gain a deep understanding of its unique and specific concept and aspects in Iranian elderly women with knee osteoarthritis may help to develop self-care competence interventions based on their specific needs.

## Methods

**Research design**: This qualitative study conducted from March to November 2020 in Mashhad (one of the largest cities in Iran). A total of 19 participants including 11 elderly women with knee osteoarthritis, 4 first degree relatives of them, and 4 medical staff were selected by the purposive sampling to provide the researcher with a sample to access specialized insight obtained from participants regarding their perceptions and experiences related to self-care competence in elderly women with knee osteoarthritis. Elderly women with knee osteoarthritis were eligible for the study based on the inclusion criteria such as being aged 65 or above, having a good mental health, ability of verbal communication, and at least a 2-year history of knee osteoarthritis. The first-degree relatives of elderly women were selected among the adults with a good physical and mental health. Inclusion criteria for the medical staff was having at least 2 years of experience in working with patients diagnosed with knee osteoarthritis. The sampling was continued until data saturation was reached, i.e. until new information was not obtained through subsequent interviews anymore and the obtained data was duplicated. The effort was made to choose people with maximum variety of self-care activities, economic, social and educational backgrounds as well as widows, married, with and without children.

**Data collection procedure**: To collect the data, in-depth and semi-structured face-to-face interviews were carried out by preparation of an audio file using audio-recording software on a mobile phone. A total of 19 interviews were carried out including 11 interviews with elderly women with knee osteoarthritis, 4 interviews with their first-degree relatives, and 4 interviews with the medical staff. The interviews were conducted in the environments where privacy could be assured and participants felt more comfortable, and, at their suggestion, in places such as the physicians' offices, physiotherapy centers, and nursing homes. To facilitate the interviews, we used an interview guide which was developed based on scientific knowledge, by the investigating team and consisted of semi-structured open-ended questions. . The questions included “What is your experience of this disease?” “How do you feel about that?” “Are you able to take care of yourself despite your disease?” The main question asked from first-degree relatives and medical staff was “What are your experiences of patients' self-care ability? During data collection and analysis, some interview questions were modified or added to generate more information on potential emerging themes. Probing questions were asked for further clarification (e.g. “What do you mean?”, “Will you elaborate further?”). Silent probes allowed participants to reflect on descriptions. The duration of the interviews ranged from 35 to 50 minutes. All the interviews were conducted in Persian by the first author, a doctoral student trained in healthcare qualitative research.

**Data analysis**: The interviews were analyzed by the conventional content analysis method. Qualitative content analysis is a widely used method for interpreting the content of textual data through a process of systematic classification, coding, and identification of patterns or themes. Data were analyzed in five steps based on method proposed by Lundman and Graneheim (18). In the first step the interviews were read through and listened to several times by the first author to gain a sense of the whole. In the second step meaning units related to the aim were identified. In the third step the meaning units were condensed and labeled and finally coded on the basis of their content. Based on the codes, sub-categories and categories were developed in the fourth step. In the fifth step the categories were carefully discussed until main categories could be identified. The MAXQDA (Version 10) was used to organize, code, and manage the data.

**Rigor of study**: The criteria proposed by Lincoln and Cuba were used for establishing trustworthiness of the study findings using member checking, integrating the data sources and method integration, endorsing the coding by the colleagues familiar with qualitative research, coding, classifying similar codes and categories, transcribing the interviews as soon as possible and peer debriefing. In addition, the researcher carefully registered the research documentations to allow an external reviewer to evaluate the study.

**Ethical considerations**: The study was carried out in accordance with the Declaration of Helsinki. The ethics committee of Tehran Islamic Azad University of Medical Sciences has approved this study by the code IR.IAU.TMU.REC.2020.170. The necessary permits were obtained from the Vice Chancellor for Research in the Islamic Azad University of Tehran to introduce the researcher to the research environment. Informed and written consent were obtained from the participants for voluntary participation in the research. Participants were told that they could withdraw from the study at any time without giving a reason and their non-participation in the study did not interfere with their treatment and medical or care process. After assuring participants about the confidentiality of information and participants' consent to audio recording during the interview process, data were collected.

## Results

The Consolidated Criteria for Reporting Qualitative Research (COREQ) checklist was used to report important aspects of this study. Demographic characteristics of the participants are shown in Table 1. From the analysis of the interviews, finally, 20 sub-categories, 9 secondary categories, and 3 main categories including “symptom management”, “personal growth” and “social cohesion” were emerged (Table 2).

**Table 1:** Demographic characteristics of the participants

Participants	Code	Gender	Age (year)	Marital status	Education	Disease duration (years)	Job	Duration of interview (mins)
**Elderly** **women with** **osteoarthritis**	1	Female	77	Widow	Diploma	10	Housewife	50
	2	Female	67	Married	Diploma	12	Chef	40
	3	Female	68	Married	Diploma	10	Member of the Board of Trustees of the Charity	42
	4	Female	81	Widow	Secondary school diploma	20	Housewife	35
	5	Female	72	Widow	Preliminary school	11	Housewife	38
	8	Female	66	Married	Uneducated	7	Housewife	35
	9	Female	65	Widow	Associate degree	35	Retired	42
	11	Female	65	Married	Preliminary school	10	Housewife	39
	12	Female	73	Widow	Uneducated	15	Housewife	35
	14	Female	79	Widow	Uneducated	14	Housewife	35
	19	Female	65	Married	Secondary school diploma	6	Housewife	37
**First-degree** **relatives**	6	Female	49	Married	Diploma	Daughter	Housewife	40
	7	Female	50	Married	B.Sc. in nursing	Daughter	Housewife	45
	10	Male	70	Married	Secondary school diploma	Spouse	Working in manufacturing workshop	35
	13	Female	52	Single	B.A. in management	Daughter	Bank teller	42
**Medical staff**	15	Male	50	Married	Specialist	15	Rheumatologist (physician)	37
	16	Male	52	Married	Specialist	20	Rheumatologist	35
	17	Male	58	Married	M.Sc.	30	Physiotherapist	45
	18	Female	29	Single	B.Sc.	5	Physiotherapist	42

**Table 2:** Main classes, subclasses and subclasses extracted from the data

Main class	Subclass	Subclasses
**Symptom** **management**	Increased physical capacity	Effective pain management - changing previous lifestyle procedures - Balance of activity and rest - Awareness of physical changes
	Improving functional power	Independence in daily activities- mobility and dynamism
	Trying to achieve vitality	Stress management – coping with illness – hopefulness
**Personal** **growth**	Spiritual Growth (Spiritualism)	Faith in The Providence and Destiny of God - Trust and Thanksgiving of God
	Getting stronger	Mastery of life –ability to take care of others
	Valuation for health	Acceptance of health behaviors – understanding the importance of health
**Social** **cohesion**	Continuity of role	Authority –ability to make decisions
	Continuation of social relations	Maintaining social relationships
	Social support	Family support – relationship with peers

*1.* **Symptoms management**: Symptoms management is accepting the inevitability of symptoms, signs, and complications among the elderly women with knee osteoarthritis and subsequently individual's effort to overcome and manage these symptoms, which include increasing physical capacity, improving functional capacity, and striving for vitality.

**1-1. Increased physical capacity**: According to the participants, the patient tries to increase her physical capacity to deal with the disease and maintain her ability by effectively managing the pain, changing previous life routines, balancing rest and activity, and being aware of the physical changes. Patients try to increase their self-care competence by using effective pain management strategies such as following the recommended treatment regimens, using distraction techniques, using painkillers, and some non-pharmacological methods, as well as accepting invasive treatments such as intra-articular injections. In this regard, a 77-year-old woman said: “*If I do not read books or newspapers, or solve a crossword puzzle, it will be difficult for me. I have a lot of entertainments, but the pain is also too much. When I do such things, I forget about the pain*”. A 67-year-old woman added: “*I tried to reduce my pain by keeping my feet warm, massaging them, tying them, and not bending as much as possible, and I even try to say my prayer in the sitting position*.”

Mobility limitations the disease create force a person to change a series of habits in life to be able to cope with daily life tasks and take care of himself/herself alone. The 52-year-old rheumatologist said: “*The most important thing is to change the lifestyle of these patients, for example, not doing hard and tedious work, using a flush toilet bowl, and using walking sticks and walkers, which improves their quality of life*.”

**1-2. Promotion of functional power**: By trying to maintain the independence in daily life activities, and the mobility and dynamism, the person tries to improve her functional power and consequently her self-care competence. The patient's constantly tries to be able to maintain her independence despite the disease, in different situations, and move towards self-regulation in various matters of life. They also try not to focus on the disease in their thoughts of the daily activities and try to act independently and stand on their own with a positive outlook to the future. In this regard, a 50-year-old woman (patient's daughter) said: "*They try to do whatever they want on their own. They even prefer to bring a glass of water by themselves despite the pain they are through. But don't ask anyone to do it. It is difficult for them to accept that now, after a lifetime, ask someone else to do their job*”.

Maintaining independence in the activities of daily living requires the ability to maintain the necessary mobility and dynamism to take care of oneself. As long as a person does not maintain her mobility and dynamism, he/she certainly cannot claim to have gained independence in carrying out the daily activities of her life. A 49-year-old woman (patient's daughter) states: “*As soon as a person walks despite the pain and does her own tasks, it is like the joints move like a car you pour the oil in its engine and it starts moving. In my opinion, the more they walk and do their job on their own, it would be much better for them*”.

**1-3. Effort for vitality**: Some people are more flexible in dealing with difficult situations. Some people possess this power and capability. Effort for vitality leads to the people growth for better thinking and more self-control. Many participants try to be flexible in difficult and stressful situations by managing stress, coping with the disease, and hopefulness. One tries to reduce the double effect of mental problems on the disease signs and symptoms to be able to manage their life and stress. In this regard, a 79-year-old woman said: “*When I am stressful and angry, I try to empty myself somehow, for example, I call my friend or sister to reduce my anger and stress. Every time I mope, my pain gets worse*”.

**2. Individual growth**: With the increase in age and subsequent overcoming of the disease, one tries to strengthen their internal structure, and achieve the ability to care for themselves. Spiritual growth (spiritualism), personal empowerment, and valuation of the health are among the subjects under study in this regard.

2-1. **Spiritual growth (spiritualism)**: For the spiritual growth sub-category, with faith in divine providence and destiny, trust, and being grateful to God, one always tries to increase the ability to endure excruciating and unbearable pain. Individual and religious beliefs are among the ways the elderly rely on to overcome the disease and its symptoms, thereby increasing her self-care competence. In this regard, a 66-year-old woman said: “*I believe that if someone has strong faith and trusts in God, s/he can somehow overcome the pain. In situations where I am in a lot of pain, I resort to the Quran. It gives me a lot of peace. It creates a state in me as if I am not in pain at all. This makes it easier for me to do my tasks*”.

**2-2. Getting stronger personally**: In such a situation, one shows himself stronger than before so that he can rely on himself, manage the affairs of life and get rid of dependence on others. Some participants suggested that they still have control over all aspects of their life. In this regard, a 68-year-old woman said: “*I feel I should be much stronger than before. I like to always stand on my own feet, serve my children, and manage my life*”.

**2-3. Valuation of health**: According to the participants in the research, the elderly person, when faced with mobility limitations caused by the disease, tries to maintain and improve her competence by accepting health behaviors and understanding the importance of health. Patients try to prevent the disease progression and its associated disabilities by observing healthy behaviors such as weight control, exercise and a specific diet. They suggest that they are unable to control their weight due to inactivity, the effects of comorbidities, or taking certain medications. They are unable to follow a regular exercise program due to lethargy and fatigue. They speak of the effect of some foods on reducing or increasing pain.

Some participants realize the importance of maintaining good health and focus on controlling the factors that affect a person's physical health when they face this disease. In this regard, a 77-year-old woman said: “*I think the foot is the main pillar of the human body because we have to do most of life activities by the aid of the foot. I could not keep my legs healthy. In my opinion, everyone should think about their own health issues from the young age so that they do not face these problems in old age*”.

**3. Social cohesion**: Elderly women with knee osteoarthritis stated that despite the limited mobility, they try to maintain their competence as before by maintaining and continuing social roles and relationships, and consider receiving social support to be effective in motivating themselves.

**3-1. Role continuity**: The elderly person, despite the mobility limitations, tries to show a sense of worth and competence by maintaining her role in the family and the ability to make decisions as before. Maintenance of the authority promotes self-confidence in the elderly and maintains a sense of power and competence in them. Elderly people, as a good mother or a good father, a good grandmother or a good grandfather, feel powerful and worthy when they see their children still need them, they can still play a role as a mother or father despite their mobility limitations, or when they play an important role in the success of their children, they are useful parents for their children, and they feel that the children are those who need the help and support of the parents. This of course can affect the level of self-care among the elderly. “*I still have to serve the children, I feel they need me*,” said a 68-year-old. This makes me stand up and get lively. I'll try not to get my house out of that boom. A 77-year-old woman added: “*I try to keep myself lively. Finally, I tell myself that although my children have grown up, they still need me and life goes on*”.

**3-2. Continuation of social relations**: Social relations are an integral part of human life. These social relations and interactions lead to gaining energy, maintaining morale and creating motivation in managing one's life. This issue may somehow affect the elderly with limited mobility. However, some seniors still try to maintain these relations despite mobility limitations.

Social interactions and interpersonal relationships can help an older person maintain good physical and mental health. Some of them try to maintain their previous social relationships, morale and authority, and they worry that the disease-based mobility limitations would affect those relationships. In this regard, a 49-year-old woman (patient's daughter) said: “*My mother is a person who becomes energetic by being with a group, if she does not go out, she will become depressed and incapacitated. My mom goes to charity on Tuesdays. Well, the same plan of going to charity changes their mood to some extent*”.

**3-3. Social support**: The elderly person receives support from her spouse, children and peers. According to the participants, this support plays an important role in maintaining morale and motivating the person, and helps to improve the person's ability to take care of himself. When faced with an illness, the family is the first source of support for the individual. In this regard, a 29-year-old physiotherapist said: “*Family support is very important so that patients does not experience stress. Now, if there is no family support, patients may not even be able to fulfill their daily tasks. For example, when a person is unable to make a meal for herself or do her daily shopping, or do other things or activities*”.

Receiving support from peers also makes a person feel that he also has the necessary abilities to manage himself and her life seeing the performance of other people like her; seeing the elderly with similar problems and receiving support from them encourage and motivate one to continue the life and affect the level of competence of the person. In this regard, a 52-year-old woman (patient's daughter) said: “*There is a special pool for patients with mobility problems, in which patients perform sports movements. My mother goes there once a week, because she talks to her peers and sees their problems, she says thank God I am not so helpless yet*”.

## Discussion

According to the results of the present study, self-care competence refers to the ability of an individual to manage the symptoms of the disease by taking advantage of personal growth and maintaining social cohesion. One strives to overcome her symptoms by increasing physical capacity, functional capacity, achieving vitality, strengthening one's internal structure with spiritual growth, becoming stronger personally and valuation of health, maintaining role continuity, maintaining social relationships, and effective social support.

In terms of the symptoms management, strategies such as effective pain management, changing previous lifestyle routines, creating a balance between the activity and rest, and awareness of physical changes can enhance self-care competence in older women with knee osteoarthritis. Pain is the most common symptom experienced by patients with knee osteoarthritis and, along with mobility disorders, often affects their ability to adapt to this progressive disease and causes debilitation in the elderly (10). Being active is a significant positive factor for self-care competence among the stay at-home elderly older women should stay as active as possible and in case of lack of physical activity, they should increase their level of regular activity in a variety of ways, with the support of others (19). The resulting physical changes can have profound effects on the lifestyle of the elderly, the relatives and society as a whole (20).

Capacity for performing activities of daily life is positively associated with self-care. Osteoarthritis can negatively affect patients' daily activities and quality of life (10). Due to pain, the level of physical activity in people with knee osteoarthritis is lower than the public. Therefore, increasing this level is necessary to improve the quality of life (21). Lack of physical activity affects self-care strategies by increasing inflammation and mild impairment (22). People who are more physically active show more independence in performing moderate to intense physical activity (23).

The self-care competence not only refers to desirable performance, but also includes cognitive and emotional components (17). One way to improve the abilities of the elderly is to manage stress. Stress management is the ability of people to cope with stress through which the individuals feel more comfortable and healthy and can better and more appropriately cope with stressful situations. Self-management competence also increases the self-efficacy and sense of cohesion in the elderly (24). Hope and life expectancy are among the biggest goals and important issues in the health care, and increasing the people's health level, especially for the elderly and the impaired (25). Some studies have implied the mental vitality. They have described mental vitality as having energy, enthusiasm and well-being, and have shown that low mental vitality creates irritability and fatigue and reduces energy to perform the daily activities. On the contrary, when mental vitality is high, there is enough energy to perform daily activities, the mood is good, and the activities are going well (26). Decreased life expectancy causes an inactive state in the elderly. In this condition, the elderly person is not able to assess different situations, her decision-making power is decreased, and she becomes defenseless against stressors. It is especially seen among the elderly women living in nursing homes who are away from their children and family. They lose all their hope over time, and this frustration weakens their problem-solving skills and makes them to evaluate their experiences negatively and incorrectly (25). In terms of the personal growth, we discussed the factors of spiritual growth (spirituality), personal empowerment, and valuation for health. On the spiritual growth (spiritualism), we raised some factors such as belief in divine providence and destiny, trust and being grateful to God. The results of studies show that faith and spirituality play an important role in dealing with the disease (7).

The model of spiritual care of the sound heart in Islam considers self-care as protection of health so that the individual can use her spiritual beliefs as a source of control in self-care. This spiritual growth helps people to discover and use their natural abilities to increase mastery and control over stressful situations and, consequently, lead to increased self-confidence, a sense of hopefulness, a positive outlook to the future and life, and a sense of control over life (27). As a coping method with positive value, spirituality can play an important and central role in filling the empty space of life, supporting the elderly, facing stress, adapting to the situation and meaning of life and death (28). Regarding the personal empowerment, we raised the factors such as mastery of life and the ability to care for others. In terms of the health evaluation, we proposed the factors such as acceptance of health behaviors and understanding the importance of health. Several strategies such as exercise, strength training and weight management help to reduce the knee osteoarthritis symptoms (13). The role of nutrition in health promotion, health maintenance, disease prevention and treatment is a key issue in the self-care. Physiological changes in old age may affect nutritional needs. Although older people may be at risk for nutritional deficiencies because they cannot meet certain nutritional needs (19), due to the inactivity caused by the disease, the patients with knee osteoarthritis gain weight which results in experiencing more severe symptoms. Weight loss is an important part of disease management. There is limited evidence to demonstrate the effectiveness of weight loss in symptom control. In interventions for with the aim of better effectively of the weight loss in controlling symptoms, it is combined with exercise interventions (23). Exercise is the foundation of non-surgical management of osteoarthritis and is recommended for all clinical cases. It is effective in reducing mobility limitations and self-care in people with osteoarthritis (22). The therapeutic benefits of exercise in people with knee osteoarthritis have been well proved and its role is similar to that of analgesics and non-steroidal anti-inflammatory drugs, though with fewer side effects and risks. Due to the common muscle weakness among people with knee osteoarthritis, the exercises that strengthen the muscle are effective in pain reduction and improving physical performance (23).

Previous studies showed that the level of authority of the elderly has increased during the old age. This increase can be observed among both genders, however, it was twice as common in women as in men. Older men more supervise the conjugal families, while older women supervise single-member and single-parent families. The number of elderly people living alone is also increasing. Over the past three decades, changes in the living arrangements of the elderly, especially older women in Iran, have led to a double burden on the elderly and to the responsibilities such as family management and meeting the needs of members (29). In terms of maintaining social relations, we raised the factors such as maintaining social relations and emotions affecting social relations. Chronic disease self-management programs, especially osteoarthritis of the knee, are effective through the social support (13). In this study, on the social support, we discussed the factors such as family support and communication with peers. Family support includes motivation, financial aid, and usual assistance, and is possible through different family members (7). Domestic studies show that older women are more dependent on their families and children. Therefore, we expect that the value changes of the family have a deeper impact on the quality of women's aging experience. Some studies have made a comparison between two lifestyles, i.e. having an independent and single life or living with family members. Most studies have mentioned the positive effects of living with family members, such as physical health and improving the quality of life of the elderly (5). Peer support usually involves the knowledge, help, and experience of peers, who can be family members, friends, or neighbors, and older people share their problems and feelings with peers. Older widows experience less emotional loneliness and social isolation after attending peer support groups (30). Deprivation of social activities make the elderly prone to the depression and increase their sense of loneliness (25).

Elderly people with knee osteoarthritis often struggle with complex diets and are heavily dependent on family support. In particular, family behaviors with a focus on self-confidence and personal success, family cohesion, and accurate responses to symptoms were associated with better self-care. In contrast, nagging, direct criticism, overprotection, blame, and distraction from symptoms associated with other subjects or activities were associated with negative patient outcomes (31). There are some limitations in the present study that need to be addressed. A limitation of current study may be recall bias since participants addressed their current experience and sometimes previous experiences also. The lack of generalizability of the findings to all older adults' patients with knee osteoarthritis in different geo-cultural contexts, due to small sample size and participants' socio-demographic and cultural characteristics, are another limitations of this study.

In conclusion, self-care competence is the necessary condition for self-care activities. According to the findings, factors such as symptoms management, personal growth, and social cohesion as dimensions of self-care competence enhance older women's competence in self-care. Due to the increasing number of self-caregiving elderly women with chronic diseases, especially knee osteoarthritis, the health and social policies for enhancing self-care competence with leading resources is a necessary.
